# Analysis of Factors Affecting Brain Metastasis in Limited-Stage Small-Cell Lung Cancer Treated With Definitive Thoracic Irradiation

**DOI:** 10.3389/fonc.2020.556634

**Published:** 2020-10-29

**Authors:** Shuting Wu, Jiezhong Wang, Wei Zhang, Jiancheng Li, Haishan Wu, Zhiyu Huang, Guangrun Zhou, Jianji Pan, Mingqiu Chen

**Affiliations:** ^1^ Department of Radiation Oncology, Fujian Cancer Hospital and Fujian Medical University Cancer Hospital, Fuzhou, China; ^2^ College of Clinical Medicine, Fujian Medical University, Fuzhou, China

**Keywords:** brain metastasis, radiotherapy, small-cell lung cancer, survival, chemotherapy

## Abstract

**Background:**

Small-cell lung cancer (SCLC) is the most lethal cancer. With the development of chemotherapy and radiotherapy, brain metastasis (BM) emerged as one most predominant treatment failure. However, the factors affecting BM have not been identified completely. The purpose of this study was to investigate the risk factors involved in the development of BM in patients with limited-stage small-cell lung cancer (LS-SCLC) following definitive thoracic radiotherapy (TRT) and to provide a reference for the planning of a clinical treatment strategy.

**Methods:**

The clinical data of patients with LS-SCLC treated with neoadjuvant chemotherapy (NAC) followed by TRT were collected and retrospectively reviewed. The factors affecting BM, BM-free survival (BMFS) and overall survival (OS) rates were analyzed statistically.

**Results:**

A total of 152 patients with LS-SCLC fulfilled the inclusion criteria were reviewed. Following TRT, 31 (20.4%) patients achieved CR, 90 (59.2%) patients reached PR, 31 (20.4%) patients maintained SD, and no patients developed PD. The OS at 1, 3, and 5 years was 80.6, 34.2, and 19.4%, respectively. Multivariate analyses indicated that the greatest dimension of primary tumor (Dmax-T) and short-term response to TRT were risk factors affecting BM. The clinical N stage (cN), greatest dimension of metastatic nodes (Dmax-N), short-term response to TRT, and adjuvant chemotherapy (AC) were identified as independent factors correlated with OS.

**Conclusions:**

Poor short-term response to TRT and huger Dmax-T were risk factors for BM. AC following TRT improved patient survival, but not decreased BM. However, due to the limitations associated with the retrospective design of the present study, further prospective clinical trials are required to confirm these conclusions.

## Introduction

Lung cancer remains the most lethal type of cancer worldwide (1), with small-cell lung cancer (SCLC) accounting for 15–20% of newly diagnosed cases. Due to the characteristics of rapid tumor growth and early dissemination (2), the prognosis of SCLC is dismal, with a 5-year overall survival (OS) rate of 2.8–7.2% (3). With intensive chemotherapy the risk of extracranial progression decreases, as a result, intracranial progression emerges as one of the main types of treatment failure (4, 5). It has been reported that the 2-year cumulative risk of brain metastasis (BM) reached up to 49% for patients with limited disease and 65% for patients with extensive disease post-treatment (6).

However, the risk factors affecting BM had not been identified so far, the optimal prevention and/or treatment of BM had not been established. In the present study, we attempted to assess the risk factors of BM by retrospectively investigating the clinical data of LS-SCLC patients treated with TRT combined with systemic chemotherapy to provide a reference for treatment strategy planning.

## Patients and Methods

### Patient Selection and Data Collection

This present retrospective study was approved by the Fujian Province Cancer Hospital Institutional Review Board (approval no. YKT-2019-020-01). All patients’ information of demographics, diagnostic and staging work-up, treatment, and treatment response were extracted from medical records and were anonymized prior to analysis.

The eligibility and exclusion criteria for the current study were summarized as follows: Primary histologically proven SCLC, good performance status [Eastern Cooperative Oncology Group performance status (ECOG PS) score ≤2], efficient pretreatment workup for tumor staging and treatment evaluation, brain magnetic resonance imaging (MRI), complete follow-up data, lack of other concomitant medical conditions that required treatment, initial treatment with TRT combined with systemic chemotherapy, clinical stage I-III (T any, N any, M0, excluding T3-4 due to multiple lung nodules that are not to be encompassed in a tolerable radiation plan) according to the 8th American Joint Committee on Cancer TNM staging system (7). Patients who survived for <1 month after TRT were considered as irradiation fatalities and were excluded from the present study.

### Treatment Characteristics

All patients were initially administrated with a ≥45 Gy dose of TRT alone or TRT combined with concurrent chemotherapy (CC) following primary neoadjuvant chemotherapy (NAC). Adjuvant chemotherapy (AC) was executed basing on patient’s treatment intention and short-term treatment response to TRT. The initiation of TRT and AC was no later than 3 weeks after completion of the previous treatment schedule.

The predominant regimens of NAC included a dual-agent combination of etoposide or irinotecan with cisplatin. Carboplatin or lobaplatin or nidaplatin was alternatively used in case of intolerance to cisplatin. Whether CC and AC used was the same drug as NAC, or a different drug was dependent on the short-term response to NAC and TRT. Adjustments to the chemotherapy time intervals and dose intensities were similar to those reported in our previous study (8).

TRT was performed using the intensity-modulated radiotherapy (IMRT) technique. The targets of TRT included the gross tumor volume (GTV) and the clinical target volume (CTV) and, after 2015, the targets only included GTV and without CTV according to the standard practice at the authors’ institution (9). The GTV including the primary tumor (GTV-T) and metastatic lymph nodes (GTV-N) were contoured on the post-NAC simulation computed tomography (CT) scans. The CTV includes the GTV and the initially involved nodal regions (but not their entire pre-NAC volume). Overall irradiation dose of TRT delivered to targets ranged from 45 to 67 Gy once daily with 1.8–2.0 Gy per fraction.

### Criteria for Treatment Toxicity and Response to TRT

The toxicities of chemotherapy and TRT were assessed according to the NCI CTC (National Cancer Institute Common Toxicity Criteria) v3.0 (10) and the RTOG (Radiation Therapy Oncology Group) criteria (11), respectively.

The short-term response of the tumor to TRT was first evaluated at the date of TRT completion and was reassessed 4–6 weeks later. The short-term response was defined as clinically complete response (CR), partial response (PR), stable disease (SD), and progressive disease (PD) according to RECIST1.1 (12).

### Surveillance and Statistical Analysis

All patient outcomes were evaluated in May 2019. The primary endpoints were BM incidence, BM-free survival (BMFS) and OS time. The OS was calculated from the date of diagnosis to the date of death or the date of the last follow-up. The BMFS was defined as the duration between the date of BM diagnosis. Patients who were censored at the last follow-up date or who had died without evidence of BM were censored for BM incidence (13).

Data were analyzed using SPSS version 24.0 (IBM Corp., Armonk, NY, USA). The survival curves were constructed using the Kaplan-Meier method and compared with the log-rank test. Univariate and multivariate analyses of the association of clinical baseline characteristics [including sex, age, ECOG, tumor location, clinical TNM (cTNM) stage, greatest dimension of primary tumor (Dmax-T), greatest dimension of metastatic nodes (Dmax-N), thoracic irradiation dose, regimens, and cycles of chemotherapy (NAC, CC, and AC) and short-term response to TRT] with OS and BMFS were performed using the Cox proportional hazards model. Confidence intervals (CIs) represented 95% lower and upper limits.

Receiver operating characteristic (ROC) curve analysis was applied to establish the cut-off value of continuous variables that were most significantly correlated with BM using the area under the curve (AUC). The variables that were statistically significantly correlated with OS or BMFS were entered in the multivariate analysis using logistic regression.

## Results

### Patient Characteristics

Between February 2012 and August 2018, a total of 152 consecutive patients fulfilled the inclusion criteria, of whom 31 (20.4%) patients achieved CR, 90 (59.2%) patients reached PR, 31 (20.4%) patients maintained SD, and no patients developed PD following TRT. The clinical characteristics of the patients are presented in **Table 1**.

**Table 1:** Clinical characteristics of patients.

	Total	non-BM	BM	P
Gender				0.901
Male	138	91	47	
Female	14	9	5	
Median age (year, range)	58 (37–88)	59 (37–88)	57 (42–72)	0.685
ECOG scoring				0.712
1	53	36	17	
2	99	64	35	
Primary tumor location				0.192
Upper right	36	21	15	
Middle right	8	6	2	
Lower right	25	14	11	
Upper left	39	28	11	
Lower left	15	13	2	
Central right	16	8	8	
Central left	13	10	3	
Clinical T stage				0.741
T1	11	8	3	
T2	56	39	17	
T3	56	34	22	
T4	29	19	10	
Clinical N stage				0.388
N0	3	3	0	
N1	7	5	2	
N2	87	58	29	
N3	55	34	21	
Clinical TNM stage				0.847
II	8	6	2	
IIIA	45	31	14	
IIIB	57	37	20	
IIIC	42	26	16	
Regimens of NAC				0.396
EP	104	65	39	
EC	29	21	8	
EL	5	4	1	
EN	4	4	0	
IP	10	6	4	
Median Cycles of NAC (range)	3 (1–6)	3(1–6)	3 (1–6)	0.553
Regimens of CC				0.247
None	90	63	27	
EP	42	22	20	
EC	13	9	4	
EL	1	1	0	
EN	2	2	0	
IP	4	3	1	
Median cycles of CC (range)	1(0–2)	1 (0–2)	1 (0–2)	0.415
Regimens of AC				0.059
None	63	45	18	
EP	63	35	28	
EC	13	8	5	
EL	3	3	0	
EN	4	4	0	
IP	6	5	1	
Median cycles of AC (range)	2 (0–4)	2(0–4)	2(0–4)	0.325
Mean dose of GTV (Gy)		57.9	58.1	0.780
Tumor response				0.026
CR	31	25	6	
PR	90	60	30	
SD	31	15	16	

AC, adjuvant chemotherapy; BM, brain metastasis; CC, concurrent chemotherapy; CR, complete response; EC, etoposide carboplatin; EL, etoposide lobaplatin; EN, etoposide nidaplatin; EP, etoposide cisplatin; ECOG, Eastern Cooperative Oncology Group scoring; GTV, gross tumor volume; IP, irinotecan cisplatin; pre-NSE, pretreatment serum levels of neuron-specific enolase; NAC, neoadjuvant chemotherapy; PR, partial response; SD, stable disease.

### Overall Survival Analysis

The median follow-up time in the entire cohort and in the surviving patients was 21 (3–82) and 32 (7–86) months, respectively. At the last follow-up, 55 patients remained alive and 97 patients had died, of whom 18 patients had succumbed to extracranial progression (including locoregional or distant recurrence) alone, 52 to BM, 6 to both, and 14 to unascertainable intracranial or/and extracranial progression; a total of 7 patients lost to follow-up and were considered as dead from unknown causes when conducting the survival analysis.

The 1-, 3-, and 5-year OS rates were 80.6, 34.2, and 19.4%, respectively (**Table 2**). Univariate and multivariate analyses indicated that cN, Dmax-N, the regimen of AC and short-term response to TRT were independent factors affecting OS (**Table 3**).

**Table 2:** OS in the entire cohort.

Subgroups	OS rate (%)						BMFS rate (%)						Actuarial rate of BM (%)				
	Case (n)	1-year	3-year	5-year	P		Case of BM (n)	1-year	3-year	5-year	P		1-year	2-year	3-year	5-year	Total
Clinical T stage											>0.05						
T1	9	—	—	—			2	—	—	—							
T2	58	94.6	47.2	28.3	< 0.001	T2 vs T3	18	77.2	68.0	62.7							
T3	56	74.8	19.3	8.0			22	67.0	49.9	49.9							
T4	29	69.0	37.9	30.3			10	67.9	62.2	62.2							
Dmax-T					0.760						0.006						
≤ 3cm	46	89.1	43.0	28.6			9	82.3	79.7	79.7			8 (88.9)	9 (100)			19.6
> 3cm	106	77.0	30.4	15.3			43	66.8	51.6	47.9			34 (79.1)	40 (93.0)	42 (97.7)	43 (100)	40.6
Clinical N stage											>0.05						
N0	3	—	—	—			0	—	—	—							
N1	7	—	—	—			2	—	—	—							
N2	87	81.3	38.1	26.4	0.010	N2 vs N3	29	74.0	61.5	61.5							
N3	55	76.2	43.3	5.7			21	64.6	51.6	51.6							
Dmax-N											0.179						
≤ 2cm	70	85.5	43.9	25.8	0.016		21	86.7	67.5	62.7							
> 2cm	82	81.6	26.3	13.9			31	68.2	55.1	55.1							
Clinical TNM stage											>0.05						
II	8	—	—	—			2	—	—	—							
IIIA	45	88.5	48.7	35.4	0.001	IIIA vs IIIB	14	77.3	64.9	64.9							
IIIB	57	77.0	22.9	11.8	0.008	IIIA vs IIIC	20	69.2	59.2	59.2							
IIIC	42	73.7	28.1		0.906	IIIB vs IIIC	16	65.9	51.2	51.2							
AC					0.025						0.560						
No	63	69.6	22.9	7.6			18	72.5	70.1	60.1							
Yes	89	92.0	41.8	19.9			34	71.3	56.7	56.7							
Tumor response					0.197	CR vs PR					0.161	CR vs PR					
CR	31	76.2	39.7	29.8	0.037	CR vs SD	6	83.0	76.0	76.0	0.009	CR vs SD	5 (83.3)	5 (83.3)	6 (100)		19.4
PR	90	82.1	40.1	25.4	0.023	PR vs SD	30	76.0	60.3	60.3	0.044	PR vs SD	21 (70)	30 (100)			33.3
SD	31	77.8	25.6	0.0			16	48.4	32.2	32.2			15 (93.8)	15 (93.8)	16 (100)		51.6
Total	152	81.9	37.3	23.2			52						41(78.8)	49(94.2)	51(98.1)	52(100)	

AC, adjuvant chemotherapy; BM, brain metastasis; BMFS, brain metastasis-free survival; CR, complete response; OS, overall survival; PR, partial response; SD, stable disease.

**Table 3:** Prognostic factors by univariate and multivariate analyses for OS and BMFS.

Prognostic Factors	OS				BMFS			
	Univariate analyses		Multivariate analyses[Backward Stepwise (Wald)]		Univariate analyses		Multivariate analyses[Backward Stepwise (Wald)]	
	P	HR	P	HR	P	HR	P	HR
		(95% CI)		(95% CI)		(95% CI)		(95% CI)
Gender	0.685	0.859			0.844	1.097		
		(0.413–1.786)				(0.436–2.759)		
Age	0.042	0.976			0.077	0.97		
		(0.952–0.999)				(0.939–1.003)		
ECOG	0.334	0.81			0.948	1.02		
		(0.529–1.241)				(0.57–1.824)		
Primary tumor location	0.609	1.027			0.414	0.942		
		(0.928–1.136)				(0.816–1.087)		
Clinical T stage	0.019	1.329			0.183	1.226		
		(1.047–1.687)				(0.893–1.683)		
Dmax-T	0.111	1.071			0.031	1.125	0.025	1.133
		(0.984–1.166)				(1.011–1.251)		(1.016–1.263)
Clinical N stage	0.003	1.683	0.015	1.621	0.117	1.427		
		(1.188–2.384)		(1.098–2.393)		(0.915–2.225)		
Dmax-N	0.003	1.175	0.052	1.119	0.257	1.082		
		(1.058–1.305)		(0.999–1.253)		(0.945–1.239)		
Clinical TNM stage	0.003	1.422			0.168	1.243		
		(1.128–1.792)				(0.912–1.693)		
NAC								
Regimen	0.262	0.904			0.435	0.902		
		(0.757–1.078)				(0.697–1.168)		
Cycles	0.98	1.002			0.635	1.057		
		(0.841–1.194)				(0.842–1.326)		
CC								
Regimen	0.78	0.974			0.959	0.993		
		(0.81–1.171)				(0.771–1.28)		
Cycles	0.376	1.159			0.149	1.393		
		(0.836–1.608)				(0.888–2.185)		
AC								
Regimen	0.03	0.793	0.043	0.801	0.252	0.861		
		(0.643–0.977)		(0.646–0.993)		(0.667–1.112)		
Cycles	0.094	0.876			0.681	1.042		
		(0.75–1.023)				(0.855–1.271)		
Dose of GTV (cGy)	0.493	1			0.722	1		
		(1–1.001)				(0.999–1.001)		
Short-term response	0.033	1.431	0.002	1.662	0.008	1.813	0.006	1.872
		(1.029–1.99)		(1.196–2.309)		(1.169–2.812)		(1.192–2.939)

AC, adjuvant chemotherapy; BM, brain metastasis; BMFS, brain metastasis-free survival; CC, concurrent chemotherapy; ECOG, Eastern Cooperative Oncology Group scoring; GTV, gross tumor volume; NAC, neoadjuvant chemotherapy; OS, overall survival.

**Figure 1:**
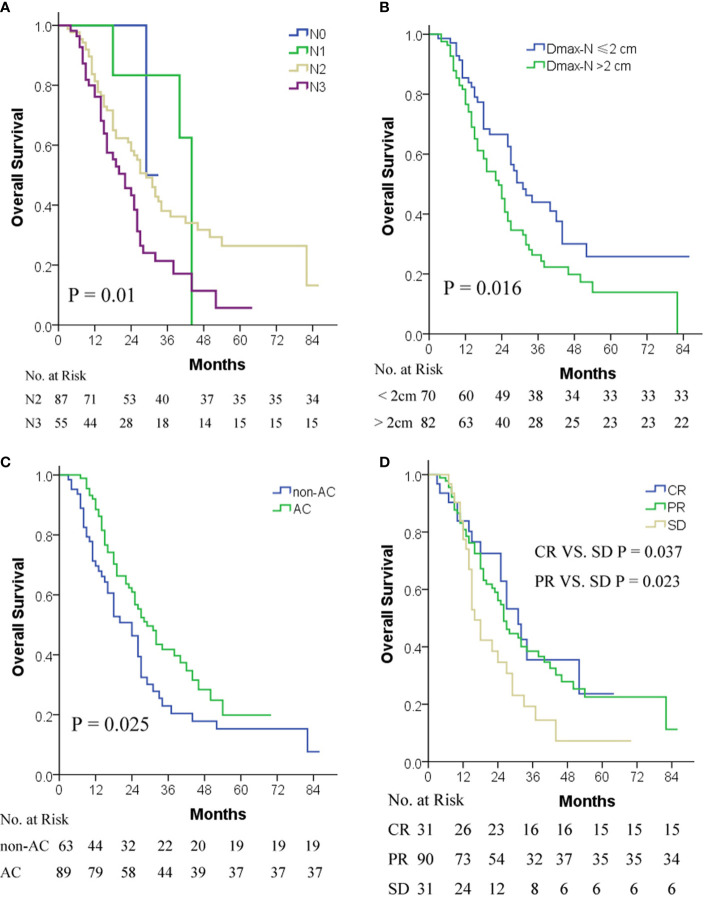
OS by cN stage **(A)**, Dmax-N **(B)**, AC **(C)**, and short-term response to TRT **(D)**. AC, adjuvant chemotherapy; cN, clinical metastatic lymph node; CR, complete response; Dmax-N, greatest dimension of metastatic nodes; OS, overall survival; PR, partial response; SD, stable disease; TRT, thoracic radiotherapy.

Compared with cN0-2 stage, patients with cN3 stage disease exhibited an inferior OS (P = 0.003), whereas the OS difference among cN0-2 stage patients did not reach statistical significance due to the limited number of patients enrolled in the present study (**Figure 1A**).

ROC curve analysis indicated that 2 cm was a cut-off value of Dmax-N in predicting OS. Patients with Dmax-N ≤2 cm exhibited a significantly superior survival compared with patients with Dmax-N >2 cm (P = 0.016) (**Figure 1B**).

Patients with AC conferred superior survival to non-AC (P = 0.025) (**Figure 1C**). However, due to the small sample size, the optimal cycles and regimens of AC were not explored in the present study.

The OS of patients with CR or PR as a short-term response to TRT was superior to those with SD, but the difference between CR and PR was not significant due to the limited number of patients enrolled in the present study (**Figure 1D**).

### Factors Affecting BM Analysis

At the last follow-up, a total of 52 (52/152, 34.2% of the entire cohort) patients encountered BM, of whom 41 (78.8%), 49 (94.2%), 51 (98.1%), and 52 (100%) patients experienced BM within 1-, 2-, 3-, and 5-years post-treatment, respectively. No new BM developed in patients who survived >5 years post-treatment.

The 1-, 3-, and 5-year BMFS rates were 71.7, 58.2, and 58.2%, respectively (**Table 2**). Different to OS, univariate and multivariate analyses demonstrated that only Dmax-T and short-term response to TRT were two independent factors affecting BM incidences and BMFS (**Table 3**).

**Figure 2:**
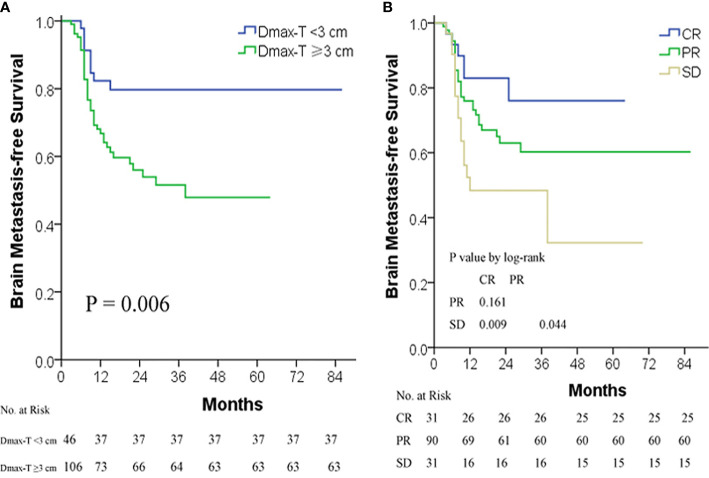
BMFS by Dmax-T **(A)** and short-term response to TRT **(B)**. BMFS, BM-free survival; CR, complete response; Dmax-T, greatest dimension of primary tumor; PR, partial response; SD, stable disease; TRT, thoracic radiotherapy.

ROC curve analysis indicated that 3 cm was a cut-off value of Dmax-T in predicting BMFS. The BM incidence and BMFS between Dmax-T ≥3 and <3 cm differed significantly (**Table 2**) (**Figure 2A**).

Similar to OS, the difference of BM incidence and BMFS was significant between CR/PR and SD but insignificant between CR and PR (**Figure 2B**).

**Figure 3:**
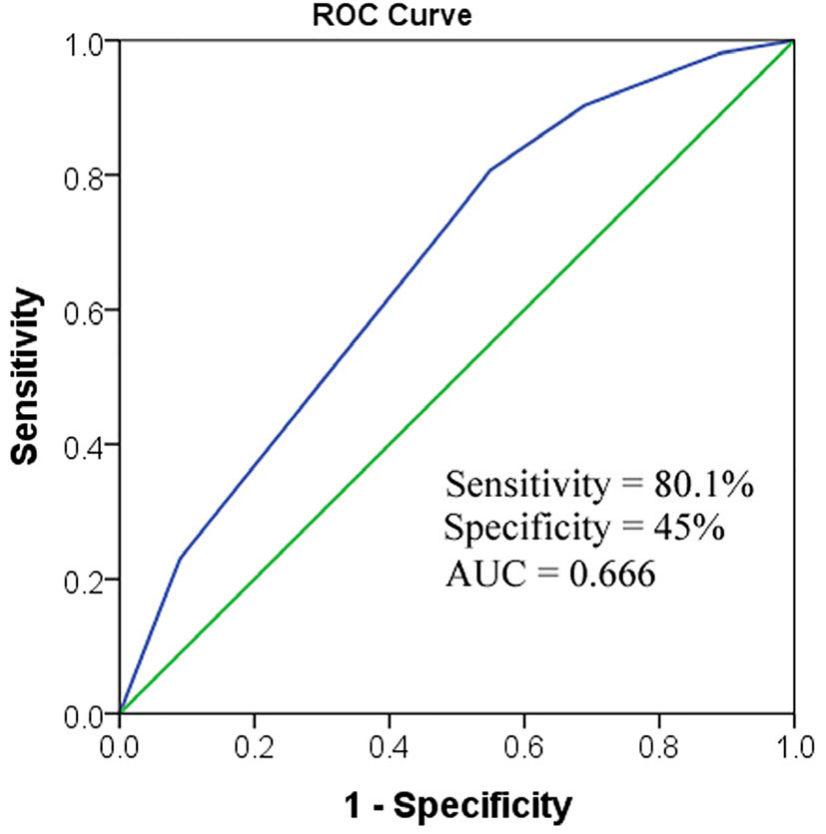
ROC to predict BM. When combining the two factors together, AUC was 0.666. AUC, area under the curve; Dmax-T, greatest dimension of primary tumor; TRT, thoracic radiotherapy.

When Dmax-T and short-term response to TRT were combined for BM prediction, the sensitivity and specificity became the maximum (sensitivity = 80.1%, specificity = 45%). The area under the curve (AUC) was 0.666 (**Figure 3**).

Compared with non-AC, AC failed to decrease BM incidence, indicating that AC can only eradicate the extracranial, but not the intracranial, occult lesions to improve patient survival (**Table 3**).

## Discussion

Gong *et al.* had conducted a study to assess the factors affecting BM in LS-SCLC patients treated primarily with surgery. They found that the pathologic stage and complete resection were independent factors affecting BM. Whereas, age, gender, NAC, AC, or adjuvant TRT following surgery were not correlated with BM (13). But, the majority of LS-SCLC patients do not qualify for surgical resection due to advanced cancer stage or concomitant diseases, and definitive TRT was recommended as part of standard treatment. While the factors related to BM in LS-SCLC patients treated with TRT had not yet been identified (14).

Zheng et al. had performed a retrospective small-sample study on factors affecting BM in LS-SCLC patients treated with TRT, which identified that T stage, neutrophil-to-lymphocyte ratio (NLR), TRT, and chemotherapy cycles were risk factors of BM (14). High T stage, high NLR, early TRT, and fewer cycles of chemotherapy were adverse factors correlated with BM. However, the nature of their retrospective study, with single-center, small-sample data, limited the reliability of the conclusions. For example, the study had concluded that earlier TRT increased the BM incidence even though TRT had improved the patients’ survival time.

Different to Zheng’s study, Farooqi et al. had recently reported a retrospective study basing on a larger scale of patients treated with definitive TRT, demonstrating that Dmax-T was the only factor associated with increased risk of BM (15). Similar to Farooqi`s study, the current study identified that Dmax-T was an independent factor correlating to BM. Patients with Dmax-T ≥3 cm developed more frequently BM than patients with Dmax-T <3 cm (19.6 vs. 40.6%).

In addition to the Dmax-T, the current study found that the short-term response to TRT was another factor of BM. The BM incidence increased sequentially in CR, PR, and SD of short-term response to TRT. High up to 51.6% of SD patients developed BM following TRT, while only 19.4% of CR patients encountered BM in the current study.

When Dmax-T and short-term response to TRT were combined for BM prediction, only 8.4% of patients with Dmax-T <3 cm and CR response to TRT developed BM post-TRT. Contrarily, the BM incidence of patients with Dmax-T ≥3 cm and SD response to TRT was as high as 57.1% in the current study.

Although Dmax-T was correlated with BM, it was not an independent factor influencing OS as shown in the current study and Farooqi’s. A reasonable explanation for the discrepancy influence of Dmax-T in BM and OS is that all TRT in the current study was conducted with IMRT technology, which achieved optimal primary tumor radiation dose coverage improving locoregional control and subsequently translated into survival benefits (16).

Numerous clinical trials in various solid tumors have confirmed that the short-term response to RT is an independent prognostic factor of survival. However, no similar studies have been conducted in SCLC to date. To our knowledge, the present study is the first to evaluate the predictive value of short-term response to TRT for survival of SCLC. It was demonstrated that short-term response to TRT was a robust factor predicting OS. Patients with CR had the best survival, followed by PR, whereas SD was associated with the poorest survival.

Chemotherapy is a cornerstone in the treatment of patients with SCLC. Several previous studies evaluated the benefit of AC following surgery in SCLC patients and reported that AC was successful in improving survival (17). Similarly, the present study demonstrated that, compared with non-AC, AC following TRT provided significant survival benefits. However, the incidence of BM in AC patients had not decreased compared to the non-AC in the current study. The reasonable explanation to the results is that AC with current chemotherapy regimens improve patient survival by eradicating the extracranial occult lesions but not the intracranial lesions due to the natural blood-brain barrier. While, limited to the number of patients enrolled in the current study, the optimal regimens and cycles of AC, the optimal sequence of AC and PCI were not explored. This is worthy of further research in future clinical trials.

In addition to the short-term response to TRT and AC, univariate and multivariate analyses in the current study indicated that the cN and Dmax-N were other two independent prognosis factors affecting OS. The higher the cN stage or the huger the Dmax-N, the worse the survival of patients. However, neither cN stage nor Dmax-N was identified correlating with BM in the current study. This finding revealed that cN stage and Dmax-N are only indicators of poor OS, but are not considered as risk factors for BM. Furthermore, the results indicated that for patients with worse N condition more intensive chemotherapy should be administrated to improve patient survival.

Besides risk factors involved BM, the interval time from the completion of TRT to BM may serve as an important reference for treatment decision making. The present study demonstrated that near 95% of BM occurred within 2 years following TRT. In other words, if patients achieved BMFS ≥2 years, the brain was no longer a risk failure site of SCLC.

## Conclusions

In conclusion, the short-term response to TRT and Dmax-T were two remarkable predictable factors affecting BM. Compared to patients with Dmax-T <3 cm and CR, patients with Dmax-T ≥3 cm and SD resulted in more frequent BM. AC following TRT improved patient survival, but not decreased BM.

There were certain limitations to the present study, such as the retrospective design from a single institution, the not sufficiently long median follow-up duration and the limited number of patients enrolled, the results of our investigation must be interpreted with caution and further prospective clinical trials are required to confirm the conclusions.

## Data Availability Statement

All datasets presented in this study are included in the article/supplementary material.

## Ethics Statement

This present retrospective study was approved by the Fujian Province Cancer Hospital Institutional Review Board (approval no. YKT-2019-020-01).

## Author Contributions

All authors listed have made a substantial, direct, and intellectual contribution to the present study, and have approved it for publication.

## Funding

This study was supported in part by grants from the Fujian Provincial All rivers run into sea of high-end talent fund, the Fujian Provincial Health & Family Planning Commission (Project Number: 2016-ZQN-32), the Fujian Provincial Department of Science & Technology (Project Number: 2017Y9079), and the Science & Technology Program of Fujian Province (Project Number: 2018Y2003).

## Conflict of Interest

The authors declare that the research was conducted in the absence of any commercial or financial relationships that could be construed as a potential conflict of interest.
